# ICE SXT vs. ICE*Sh95*: Co-existence of Integrative and Conjugative Elements and Competition for a New Host

**DOI:** 10.1038/s41598-019-44312-1

**Published:** 2019-05-29

**Authors:** Gisela Parmeciano DI Noto, Andrés Iriarte, María Soledad Ramírez, Daniela Centrón, Cecilia Quiroga

**Affiliations:** 1 Universidad de Buenos Aires, Consejo Nacional de Investigaciones Científicas y Tecnológicas, Instituto de Investigaciones en Microbiología y Parasitología Médica (IMPAM), Facultad de Medicina, Buenos Aires, Argentina; 2 Laboratorio de Biología Computacional, Departamento de Desarrollo Biotecnológico, Instituto de Higiene, Facultad de Medicina, Universidad de la República, Montevideo, Uruguay; 3 Center for Applied Biotechnology Studies, Department of Biological Science, College of Natural Sciences and Mathematics, California State University Fullerton, Fullerton, California USA

**Keywords:** Data processing, Genome assembly algorithms, Microbiology, DNA recombination

## Abstract

Integrative and conjugative elements (ICEs) are mobile genetic elements that contribute to horizontal gene transfer. The aim of this work was to study different types of ICEs in clinical isolates of the emergent pathogen *Shewanella* spp., to compare their transfer efficiency and their ability to integrate a new host. Here we show that 3 out of 10 clinical isolates contained an ICE. Two of these elements were similar to ICEs from the SXT/R391 family and the other one was similar to ICE*Sh95*, a hybrid platform. Mating assays showed that these elements co-exist for several generations in the same host. Furthermore, transfer rates and competition assays between ICE*Sh95* and ICE*Sh392*, an SXT-like element, suggest that the latter has evolved into a well-oiled machine that efficiently spread to different bacteria. Our results provide strong evidence of the role that ICEs play in the dissemination of genetic traits in nature and the implications that they have in the global threat of antimicrobial resistance.

## Introduction

Integrative and conjugative elements (ICEs) are large genomic platforms capable of self-transferring. These elements play a key role in the genetic exchange between bacteria via horizontal transfer events^1–3^. ICEs encode in their platform the mechanisms of excision, integration, self-transfer, and regulation. Furthermore, they contain accessory modules identified as variable regions or hot spots (HS), which are commonly unique for each element^1–3^. The genetic information found in these regions can confer adaptive advantages to their host, such as antibiotic and heavy metal resistance, restriction modification systems, DNA repair systems or toxin, and antitoxin systems^1–5^. ICEs can be classified in different families^2,3^. Among them, the SXT/R391 family has been extensively studied and their members are widely disseminated among gamma-proteobacteria^2,3^. Recently, the criteria for classifying ICEs of the SXT/R391 family was redefined in four types based on their insertion site, structure, prevalence, and distribution in bacterial species^6^. While type I includes all ICEs integrated at the 5′ end of *prfC* gene found in *Enterobacteriaceae* and *Vibrionaceae*, types 2, 3 and 4 ICEs are integrated at the 3′ end of the tRNA-Ser gene and are exclusively found in *Vibrio* species^6^.

Different ICEs from the SXT/R391 family have been found in environmental and clinical strains of *Shewanella*^7–11^, a gram-negative rod that thrives in aquatic niches^12^. In recent years there has been an increase in the reports on *Shewanella* spp. isolated from clinical samples establishing it as an emerging opportunistic pathogen^13–17^. This bacterium is also known for its potential application in bioremediation, its versatile metabolism, and its genetic plasticity^12^.

We have previously described a hybrid and fully active ICE in the clinical isolate *Shewanella* sp. Sh95, named ICE*Sh95*^10^. This element contained a multidrug resistant (MDR) integron platform harboring two gene cassettes that confer resistance to trimethoprim and quaternary ammonium compounds. Moreover, we showed that ICE*Sh95* has the core genes conserved among members of the SXT/R391 family and it was integrated at the gene *pabA*, a para-aminobenzoate synthase involved in folate biosynthesis.

This work aimed to characterize different types of ICEs found in clinical isolates of *Shewanella* spp. by assessing their transfer efficiency, maintenance, and activity. Our study evidenced that *Shewanella* spp. can carry different ICEs platforms, which in turn can spread to other hosts playing an important role in horizontal gene transfer (HGT). Furthermore, we suggest that the higher occurrence of ICEs from the type I SXT/R391 family in nature instead of hybrid elements is the result of an efficient transfer machinery. This study provides additional information on the impact of ICEs in bacterial genome evolution and on their contribution in the dissemination of antibiotic resistance determinants from environmental niches to clinical settings.

## Results and Discussion

### Identification of ICEs from *Shewanella* clinical isolates

We looked for ICEs from the SXT/R391 family and ICE*Sh95*-like in 10 clinical strains, which were obtained from a public hospital of Buenos Aires, Argentina^10,18,19^. The screening was done by PCR using specific primers to detect two conserved regions^6,20^, the origin of transfer, *oriT*, and the *setR* gene. Our results showed that 3 out of 10 isolates contained an ICE corresponding to strains *Shewanella* sp. Sh31, *Shewanella* sp. Sh82, and *Shewanella* sp. Sh392^10^. Identification of each ICE was done by PCR amplification using specific primers for the *xis/int* module of ICEs SXT/R391 and ICE*Sh95*. As a result, we observed that ICE*Sh31* from *Shewanella* sp. Sh31 contained the *xis/int* module found in ICE*Sh95*, whereas *Shewanella* sp. Sh82 and Sh392 (ICE*Sh82* and ICE*Sh392*, respectively) were positive for an SXT/R391-like ICE^6,10,21^. We then searched for the integron described in ICE*Sh95*^10^, located in the variable region HS3, in the strains bearing an ICE using specific primers. Similar to ICE*Sh95*, ICE*Sh31* contained an MDR integron carrying the *dfrA15* gene cassette, which confers resistance to trimethoprim.

The common features found between ICE*Sh95* and ICE*Sh31* were consistent with phylogenetic studies previously reported^10^, in which it was shown that strains Sh31 and Sh82 are closely related to Sh95, whereas strain Sh392 belonged to the *S*. *algae***/***S*. *haliotis* lineage^10^. Taken together, these results confirmed that *Shewanella* spp. can acquire either an ICE of the SXT/R391 family (Sh82 and Sh392 strains) or a hybrid element such as ICE*Sh95* (Sh95 and Sh31 strains) regardless its source. Thus, we observed that ICEs could contribute to the evolution of bacterial pathogens to extensive drug resistance by acting as vectors spreading key traits to different hosts.

### Transfer ability of ICEs in *Shewanella* spp

In order to evaluate the ability of each ICE to self-transfer to other bacteria we did a conjugative assay and looked for the excised forms by PCR. Our results showed that ICE*Sh31* and ICE*Sh392* were able to form circular intermediaries and self-transfer to other hosts, whereas ICE*Sh82* did not exhibit any transfer activity nor its circular form was detected, which suggest that it may have lost its ability.

**Figure 1:**
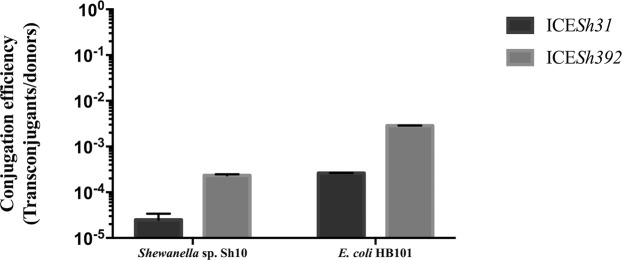
Conjugation efficiencies of ICE*Sh31* and ICE*Sh392*. Efficiencies were scored as transconjugants/donors, ICE*Sh31* is depicted with black bars and ICE*Sh392* with grey bars. Data shown are the mean ± SD (n = 3).

ICE*Sh31* was able to transfer to *Shewanella* sp. Sh10 with a frequency of 2.5 × 10^−5^ (±0.90 × 10^−5^) and to *E*. *coli* HB101 with a frequency of 2.62 × 10^−4^ (±0.37 × 10^−5^). On the other hand, ICE*Sh392* showed transfer rates of 2.33 × 10^−4^ (±1.53 × 10^−5^) for *Shewanella* sp. Sh10 and 2.86 × 10^−3^ (±0.34 × 10^−4^) for *E*. *coli* HB101 (Fig. 1).

Previously, we have reported that ICE*Sh95* was able to transfer to other *Shewanella* spp. with a frequency of 2.85 × 10^−3^ (±2.15 × 10^−3^) and to *E*. *coli* with a frequency of 1.04 × 10^−5^ (±0.60 × 10^−5^)^10^. Taken together, these results confirm that both ICE*Sh392* and ICE*Sh31* efficiently participate in the genetic exchange and genome diversity of different bacteria. Since ICE*Sh392* is an active mobile element and its recombination module is similar to those from the SXT/R391 family we decided to sequence the host genome and further study this ICE.

### ICE*Sh392* is similar to members of the type I SXT/R391 family

*De novo* assembly of the genome of strain Sh392 resulted in 4,803,735 bp with an N_50_ contig size of 96,236 bp (max. length 367,520 bp) and a G + C content of 52.9%. Sequence annotation using RAST led to the identification of 4,200 coding genes and 123 tRNA genes^22^. We calculated the average nucleotide identity (ANI) to estimate the proximity between species, which resulted in 98.38% (±1.42%) identity to the closest relative *S*. *haliotis* JCM 14758 (cut-off: 95%)^23^, now proposed *S*. *algae*^24^. It is worth to mention that *S*. *algae* is considered the causative agent of skin and soft tissue infections, bacteremia and osteomyelitis^14,15,17^.

**Figure 2:**
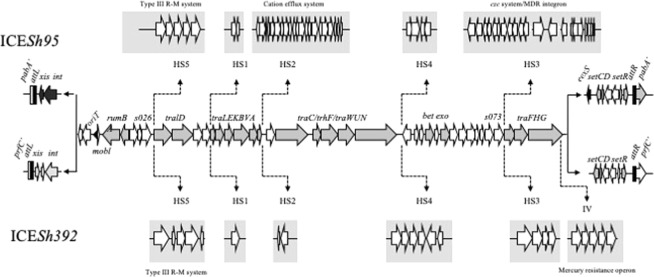
Comparison of ICE*Sh95* and ICE*Sh392* genetic architecture. Structural core genes shared between both ICEs (light gray and white) are shown in the middle of the figure. Hot spots for each ICE are depicted in grey boxes. The attachment sites are depicted in black (*attL* and *attR*), the *xis/int* module of ICE*Sh95* and the *eexS* gene are in black.

We then focused in the region comprising ICE*Sh392*, which revealed that it contained the complete backbone of the ICE of the family SXT/R391 (Fig. 2). Its G + C content was 47.31%, which is lower than the average G + C content of *S*. *algae* Sh392 genome (52.9%). This is in line with Cury *et al*. (2017), who reported that ICEs are elements rich in As and Ts. Furthermore, we did a phylogenetic analysis using the *setCD* operon and *setR* to identify the closest relative of ICE*Sh392*. The concatenated tree showed that these elements, *setCD* and *setR*, clustered with those from ICE SXT_MO10_ and R391, which indicates that ICE*Sh392* belonged to the type I SXT/R391 lineage (Fig. S1).

The analysis of all ICE*Sh392* modules showed that its integrase shared 98% amino acid identity with the integrase of SXT_MO10_ while the other core regions also showed strong similarities (>95% nucleotide identity). In addition, we identified all HS and variable regions from this element (Fig. 2). In HS5 we found a putative type III restriction and modification system with poor nucleotide identity to the one found in ICE*Sh95;* whereas, the variable region IV contained a mercury resistance operon, a common feature of most ICE reported in *Shewanella* spp.^8–11^. Noteworthy, ICE*Sh392* did not encode antimicrobial resistance determinants.

### Insertion site of ICE*Sh392*

Analysis of the insertion site of ICE*Sh392* showed that this element was located at the *prfC* gene similarly to type I SXT/R391 family members (Fig. 2)^4^. We examined the extremities of ICE*Sh392* and found the putative sequences for the *attL* and *attR* attachment sites (5′-ATCATCTCTCACCCGGA-3′ and 5′-ATCATTTCTCACCCTGA-3′, respectively). PCR amplification of the exconjugant product and sequence analysis revealed that the attachment site *attP* was 5′-ATCATCTCTCACCCTGA-3′, and based on *attP*, *attL* and *attR* sequences we were able to infer that the *attB* sequence was 5′-ATCATCTCTCACCCGGA-3′. Alike other SXTs, our ICE also integrated at the gene *prfC* without altering the open reading frame, which shows that these elements have a bias towards that gene.

### Co-existence and competition of ICEs

We wondered whether an ICE of the SXT/R391 family and an ICE*Sh95*-like could co-exist. Based on the entry exclusion group *eex* gene^25^, we were able to determine that ICE*Sh95* belonged to type S group (gene *eexS*)^10^. On the other hand, we could not identify a recognizable *eex* gene in ICE*Sh392*. This gene is commonly located downstream of the gene *traG*, instead we found a mercury resistance operon (*mer*) at that locus. Since no exclusion gene was detected, we proceeded to test the ability of the host to acquire and maintain both ICEs using strains Sh95 and Sh392 as donor and recipient cells. We performed mating assays using *Shewanella* sp. Sh95 as recipient strain and *S*. *algae* Sh392 as donor strain and vice-versa. We selected the different transconjugants using trimethoprim (150 μg/ml) and meropenem (6 μg/ml) and checked for the presence of either ICE by PCR using specific primers for the respective *xis/int* modules (Fig. S2A). Our results showed that these ICEs co-exist in the same strain regardless of their presence in the recipient cell (Fig. S2A). This suggests that none of these elements exclude themselves. Moreover, we assessed the stability of these elements after serial growth in the same host and we observed that both ICEs were present after 384 generations (Fig. S2B). Since entry exclusion is a key feature for ICEs survival in a new host^25^, the absence of a known *eex* gene in ICE*Sh392* could reflect a major advantage for its dissemination to organisms already harboring an ICE.

As various ICEs co-exist in a niche, we wanted to expose different ICEs to a single recipient and evaluate their efficiency to invade a new cell. We did a competition assay using *Shewanella* sp. Sh95 and *S*. *algae* Sh392 as donor strains, and *E*. *coli* HB101 as the recipient strain. We determined the quantity of each ICE in the recipient cell (*E*. *coli*) after mating by qPCR. We used primers IntSXTqPCR-F and IntSXTqPCR-R to quantify the integrase gene from ICE*Sh392*, primers Int95qPCR-F and Int95-R-qPCR for the integrase gene from ICE*Sh95* and primers traV-F and traV-R for the gene *traV* from both ICEs. We obtained the *Ct* values from three independent assays for a specific gene from ICE*Sh95*, *int*_Sh95_ (33 ± 0.85), a specific gene from ICE*Sh392*, *int*_Sh392_ (27.7 ± 1.05), and a gene conserved in both ICEs, *traV* (22.3 ± 1.07). We calculated the normalized ratio for each ICE taking into account the respective primer efficiencies. As a result, we observed that ICE*Sh392* was 58 times more efficient than ICE*Sh95* as a self-transfer element.

In order to evaluate that these ICEs maintained their ability to re-transfer once inserted in a new genome we did a mating assay using *Shewanella* sp. Sh95 carrying both ICEs as a donor strain and *E*. *coli* HB101::pCR2.1 as recipient strain. Our results showed that ICE*Sh392* was able to transfer successfully to *E*. *coli* but no positive results were detected for ICE*Sh95* (Fig. S2C). This could be due to the higher conjugation efficiency showed by ICE*Sh392*. On the other hand, we observed that ICE*Sh95* was able to self-transfer from strain Sh392 carrying ICE*Sh392* to *E*. *coli* (data not shown). Furthermore, to confirm that there was no cross-complementation between ICEs, we assessed the individual re-transfer abilities of each element. We incubated *E*. *coli* HB101 carrying either ICE*Sh95* or ICE*Sh392* with *Shewanella* sp. Sh10 and observed that both ICEs retained their ability to conjugate to a new host (Fig. S3A–C).

Taken together, our data suggest that both ICE are active elements capable to re-transfer regardless of the host or the presence of other genomic islands. Furthermore, we propose that type I SXT/R391 ICEs are more efficient mobile elements, which is consistent with their prevalence reported in nature^2,6,10,26^.

**Figure 3:**
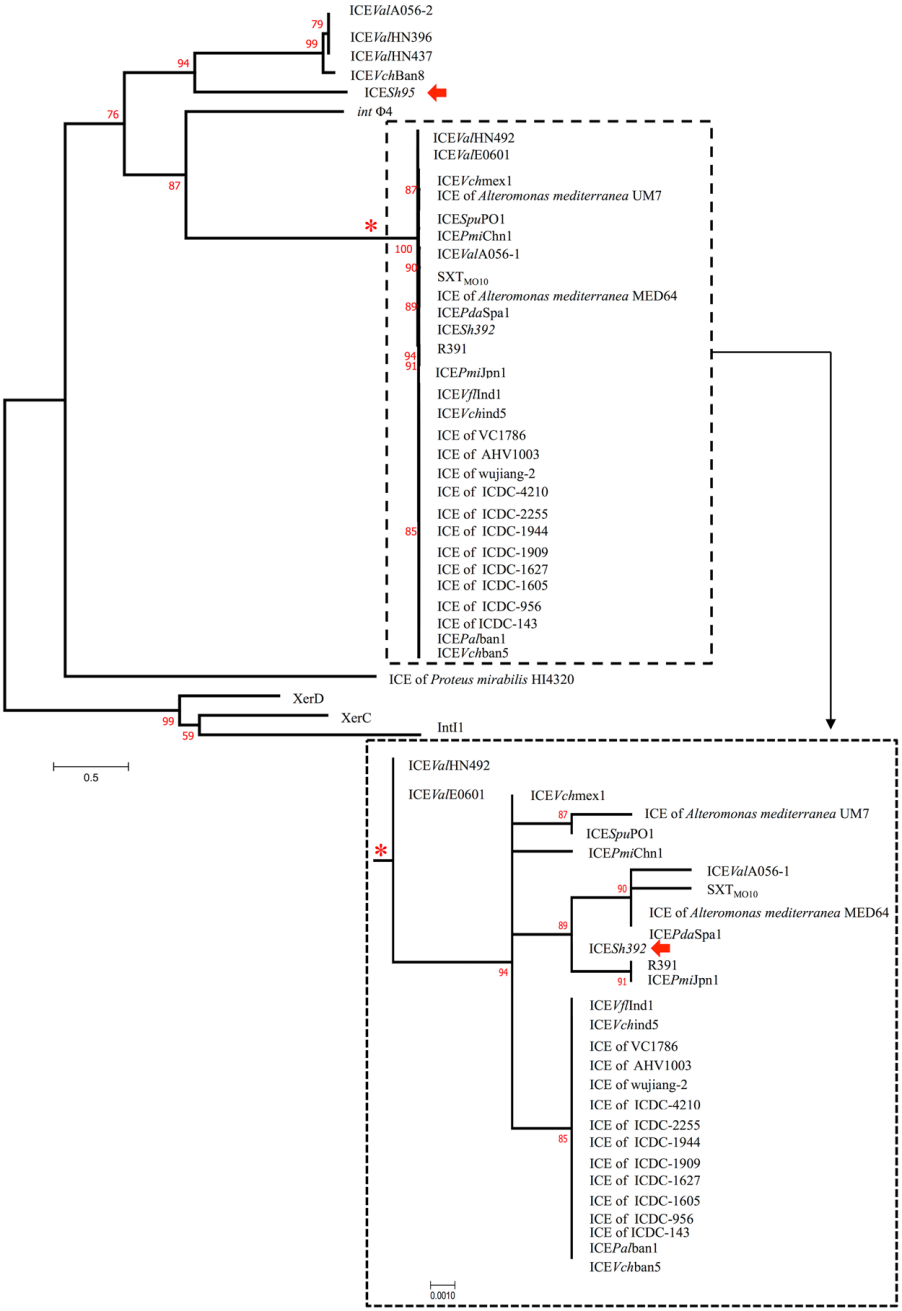
Phylogenetic tree of ICE integrases using maximun likelihood algorithm. ICE*Sh95* and ICE*Sh392* are marked with red arrows. The subcluster marked with an asterisk is presented at a larger scale.

Since the ICE integrases play a major role during the transfer process, we did a phylogenetic analysis including representatives of all known platforms. The integrase of ICE*Sh392* clustered with those from ICE SXT_MO10_ and R391. This data together with ICE*Sh392* sequence analysis (QFDC00000000) and the concatenated *setCD*/*setR* tree (Fig. S1) confirm that this element is a type 1 SXT/R391 member. On the other hand, the integrase from ICE*Sh95* was closely related to Int from type 2 and 3 SXT/R391 ICEs, which have a different integration module and are integrated at the 3′ end of the tRNA-Ser gene^6^ (Fig. 3). The differences observed between other SXT/R391 ICEs and ICE*Sh95* indicate that the latter was subjected to a distinct recombination event that originated a unique hybrid platform^10^.

Despite ICE*Sh95* is less efficiently transferred to a new host, it has evolved into a multidrug resistant platform. Taking together our results and those reported for other SXT/R391 ICEs^2,6,11^, we can infer that ICE*Sh392* from *S*. *algae* might adapt and acquire multidrug resistance genes upon antibiotic pressure.

SXT/R391 ICEs have evolved into well-oiled and highly efficient elements that either have or can recruit antimicrobial resistance genes in their platforms. Elements such as ICE*Sh392* represent a troublesome scenario in the antimicrobial resistance field, where we can foresee the evolution of opportunistic bacteria acquiring mobile platforms that can carry resistance genes or gain novel determinants, and subsequently disseminate these traits into a new niche.

### Concluding remarks

The comparative analysis of ICEs found in *Shewanella* spp. evidenced the plasticity of these mobile genetic elements, making them a potential threat facing the problem of multidrug resistance and exposing their adaptability. This report demonstrates that different ICEs can co-exist in a single strain and evaluates their ability to compete for a new host providing compelling information on the significant spread of ICEs SXT/R391 in nature.

Our results suggest that the ICEs studied here contribute to the evolution and the exchange of the genetic information between aquatic niches and nosocomial environments, where the antimicrobial pressure play a key role in the selection and survival of these bacteria.

## Methods

### Strains, plasmids and general molecular techniques

Clinical strains of Shewanella spp., including Shewanella sp. Sh9, Shewanella sp. Sh10, Shewanella sp. Sh31, Shewanella sp. Sh47, Shewanella sp. Sh74, Shewanella sp. Sh78, Shewanella sp. Sh82, Shewanella sp. Sh117, Shewanella sp. Sh256 and Shewanella sp. Sh392, the strain harbouring the ICESh95 as control, Shewanella sp. Sh95^10,18,19^ and *Escherichia coli* HB101 were grown at 37 °C in Luria-Bertani medium (10 g/l tryptone, 5 g/l yeast extract and 10 g/l NaCl in dH_2_O) with shaking at 200 rpm. Total DNA extraction was done using the Wizard Genomic DNA Purification kit (Promega). All PCR reactions were done using 1 U of Taq DNA polymerase (InBio Highway) in 1× Taq buffer (InBio Highway) supplemented with 2 mM MgCl_2_, 0.14 mM dNTP mix and 0.4 mM of each primer in a final volume of 50 μl. The PCR conditions were 3 min at 95 °C, 35 cycles of 30 s at 95 °C, 30 s at the appropriate annealing temperature and 1 min at 72 °C, followed by a final extension of 5 min at 72 °C. Amplification of the 16s rRNA gene of clinical isolates of Shewanella spp. was done using specific primers FD2 and RP2^27^. All positive PCR amplification products were confirmed by sequencing. Occurrence of SXT-ICE family in Shewanella genus was determined by PCR amplification of the origin of transfer (*oriT*) with primers oriT-SXT-F (5′-TGTATCCCTTGTCAGGTATG-3′) and oriT-SXT-R (5′-ACCCCAAAAGCCAAAACCAC-3′) and the setR gene with primers setR-SXT-F (5′-TACTGGTTACTGGCTTTCA-3′) and setR-SXT-R (5′-CATCAACTCAAAACAGCAA-3′). Detection of the excised form of ICE*Sh392* was done by using ICE-3′ end-F (5′-GTGAACAGAAGTACCTAAA-3′) and primer Sh392-5-end-F (5′-ATAACTCTAGGTCCAGTAC-3′). Characterization of the insertion site was done by PCR using primers ICE-3′ end-F (5′-GTGAACAGAAGTACCTAAA-3′), pabA-R (5′-CTCTTGCCCTAACTGCT-3′), primer pabAEc-R (5′GCACGCTGGTAATGAGTCAG-3′), primer prfCSh95-R (5′-GCGTTTCCGAATAACAGCA-3′) and prfCEc-R (5′-GAGAAATAATGGCAAAAGTG-3′). The *xis/int* module was detected using primers Xis-ICE-F (5′-GTTGAGTATATGATGCTCTGG-3′) and int-ICE-R for ICESh95-like elements (5′-CAAGTGGTCCGAAAACTACA-3′) and IntSXT-F (5′-AGACTCTGCCGGTAAGCAAG-3′) and IntSXT-R (5′-GCAACTTCTTGCGTCGTGAT-3′) for SXT-like elements. For HS3 characterization we used the following primers: inti9-Sh95R (5′-TTGAGTAGACGCCGTAACT-3), Sh95-GII-IntF (5′-TTACAGCAGGCATGGAAACA-3), Sh95-GII-IntR (5′-CGCCATTGCCGCCAATAG-3′), dfrA15-F (5′-CTATCACTAATGGCAGCA-3′), dfrA15-R (5′-GAACGAGTTACAACGGCA-3′), TraF-ICESh95-R (5′-GAACCAGGCAGCACTGA-3) and IS4-ICE-F (5′-ACCGACTTTCCGCACCTGAT-3′). Lastly, for qPCR assays we used the following primers: IntSXTqPCR-F (5′-GAGTTCGTTTGCCTTTCTTAC-3′), IntSXTqPCR-R (5′-ACTGTCTTCGTGAGGTTTCG-3′), Int95qPCR-F (5′-CGTACCATTATCCAAACAAGC-3′), Int95-R-qPCR (5′-TGCCGTACCCCT GAATCCA-3′), traV-F (5′-CAGTCGGCTCAACCTTATTG-3′) and traV-R (5′-ATCAGATTGTGAATACCCAG-3′).

### Mating assay

Mating assays were carried out on LB agar plates. Strains Sh95 (Tmp^R^), Sh31 (Tmp^R^), Sh82 (Tmp^R^) and Sh392 (Tmp^R^) were used as donor strains, while *E*. *coli* HB101::pCR2.1 (Kan^R^) and *Shewanella* sp. Sh10::pCR2.1 (Kan^R^) were used as recipient strains. Donor and recipient strains were diluted from saturated overnight cultures into 10 ml and grown for 7 h at 37 °C. The cells were harvested by centrifugation, poured onto LB agar plates and incubated at 37 °C for 18 h. The cells were scraped off the mating plates and serial dilutions were plated onto LB agar with the specific antibiotic to select for donor, recipient or transconjugant cells. Frequency of transfer was expressed as the number of transconjugant cells per donor cell in the mating mixture at the time of plating. Transconjugants obtained with both recipient strains were checked for the presence of ICE*Sh95* and SXT by PCR with the original recipient strains used as negative controls.

Co-existence assays were done using strains Sh95 and Sh392 as donor and recipient strains and transconjugant cells were tested by PCR with specific primers for each *xis/int* module. Re-transfer assay was done using *Shewanella* sp. Sh95::ICE*Sh392* as donor and *E*. *coli* HB101 as recipient strains. Transconjugant cells were tested by PCR for each *xis/int* module as described before. To evaluate the transfer efficiency of both ICEs, we did a conjugative assay using strains Sh95 and Sh392 as donors strains and *E*. *coli* HB101::pCR2.1 as recipient strain. Conjugative assays were done as described above using both donor strains in each assay so that both ICEs compete for the recipient (ratio 1:1:1). The cells were scraped off the mating plates and serial dilutions were plated onto LB agar with kanamycin (50 μg/ml) to select for the transconjugant cells. Lastly, we did a total DNA extraction of the transconjugant cells and the controls using the Wizard Genomic DNA Purification kit (Promega) and analyzed the amount of each ICE by qPCR.

### Real Time PCR assays

Real-time quantitative PCR assays were designed to measure the transfer rate of the elements SXT or ICE*Sh95-*like to *E*. *coli* HB101 and evaluate their transfer efficiency. To measure the rates of each element we calculated the amount of each integrase of ICE*Sh95* and ICE*Sh392*, which were normalized with the amount of chromosomal DNA in each sample using the conserved gene *traV*, encoded in both ICEs (adapted from ref.^21^). Briefly, each primer set was tested at different concentrations (5–30 pmol/μl) to optimize their performance. The StepOnePlus Real-Time PCR System was used to quantify the increase in fluorescence emission during PCR that resulted from the binding of the dye EVA Green to double-stranded DNA. Each 20 μl reaction mixture contained 4 μl of 5x HOT FIREPol® EvaGreen® qPCR Mix Plus (ROX) (Solis BioDyne), 5 pmol/μl of each primer and 8 ng of the DNA template. All qPCR products were electrophoresed on 1.2% agarose gels to detect the presence of a single band. We performed three independent assays. A standard curve was generated for each gene by using the donors DNA (*Shewanella* sp. Sh95 and *Shewanella* sp. Sh392) as the template and by plotting the *C*_*t*_ values as a function of the concentration of DNA in order to calculate the PCR efficiencies (*E*) as described elsewhere^21^. The efficiency of *intSh*95 was 2.00, *int*SXT was 1.90, and *traV* were 1.98 and 2.02 (with both donors, Sh95 and Sh392 respectively). We calculated the relative ratio of each *int* based on the *E* and the *C*_*t*_ deviation (Δ*C*_*t*_) of the transconjugant sample compared with each donor DNA (control samples). Each value was normalized by the calibrator sequence (*traV* gene) by using the following equation:

$${{\rm{R}}}_{int}=[{({{\rm{E}}}_{int})}^{{\rm{\Delta }}Ct(int)}]/[{({{\rm{E}}}_{traV})}^{{\rm{\Delta }}Ct(traV)}]$$

where *E*_*int*_ is the PCR efficiency of the *int* site tested (*int*_Sh95_ or *int*_SXT_), *E*_*traV*_ is the PCR efficiency of the calibrator sequence (*traV* gene), Δ*C*_*t*(*int*)_ is the difference between the *C*_*t*_ value of the donor strain and the *C*_*t*_ value of the transconjugant strain [*C*_*t(Sh95)*_ − *C*_*t(sample)*_] for the *int*_Sh95_ gene tested or [*C*_*t(Sh392)*_ − *C*_*t(sample)*_] for the *int*_SXT_, and Δ*C*_*t(traV)*_ is the difference between the *C*_*t*_ value of the donors strains and the *C*_*t*_ value of the recipient strain [*C*_*t(Sh95 or Sh392)*_ − *C*_*t(sample)*_] for the calibrator sequence.

### Genome sequencing, assembly and annotation

Total DNA of *Shewanella* sp. Sh392 was obtained using the Wizard Genomic DNA Purification kit (Promega). A draft sequence of this genome was developed using Illumina MiSeq at the Argentinian Consortium of Genomic Technology (ACGT). A total of 9938334 high-quality paired-end reads were produced, with an average insertion size of 436 bp. De novo assembly was performed with the SPAdes assembler version 3.6.2^28^. 4900160 pairs of reads plus 15837 unpaired reads, 98.8% of the generated reads, were assembled resulting in mean nucleotide coverage of 332 (and a k-mer coverage of 117). Corrected reads showed an average length of 164 bp. This Whole Genome Shotgun project has been deposited at DDBJ/EMBL/GenBank under the accession QFDC00000000.

### Phylogenetic analysis

ICEs integrases protein sequences were downloaded from GenBank and aligned using Clustal Omega v1.2.1^29^. The datasets comprise 46 amino acid sequences of various integrases of representative ICEs and other integrases as outgroups, such as XerD, XerC, IntI1 and P4 Int (Table S1). SetC, SetD and SetR protein sequences were downloaded from GenBank and independently aligned using Clustal Omega v1.2.1^29^ (Table S2). The alignments were concatenated and the final dataset comprises 34 sequences including 492 amino acid positions. A maximum likelihood phylogenetic analysis was done for each dataset by means of the PHYML v3.1 software^30^. The LG + G model was used^31^, with 8 substitution rate categories and 5 random starting trees. The default SH-like test^32^ was used for branch support assessment.

### Accession codes

The genome of *Shewanella* sp. Sh392 was deposited in GenBank as accession number QFDC00000000.

## Supplementary information

Supplementary information
