# Supplementary material for: ICE SXT vs. ICESh95: Co-existence of Integrative and Conjugative Elements and Competition for a New Host
Source: Sci Rep. 2019 May 29;9:8045. doi: 10.1038/s41598-019-44312-1 (PMC6541609; doi:10.1038/s41598-019-44312-1)
Supplement: Supplementary file 1 — Supplementary information [file 41598_2019_44312_MOESM1_ESM.pdf]

ICE SXT vs. ICESh95: Co-existence of Integrative and Conjugative Elements and Competition for a New Host

Gisela Parmeciano Di Noto1, Andrés Iriarte2, María Soledad Ramírez3, Daniela Centrón1, Cecilia Quiroga1*

Table S1.
Integrases or recombinases with their corresopnding accession numbers used for phylogenetic analysis.

Elements	Protein ID	Accession number
SXT	AAL59748.1	AY055428
R391	AAM08088.1	AY090559
ICE of *Alteromonas mediterranea* MED64	AGP81066.1	NC_023045
ICE of *Alteromonas mediterranea* UM7	AGP96682.1	NC_021713
XerC	ALI38512.1	NC_000913.3
XerD	AIZ90072.1	NC_000913.3
IntI1	ACB87555.1	NC_007100.1
Phage P4	CAA35897.1	NC_001609.1
ICE*Pal*ban1	ACV96081.1	GQ463139.1
ICE*Pda*Spa1	CAQ34932.1	AJ870986.2
ICE*Pmi*Chn1	ALP69229.1	KT962845.1
ICE*Pmi*Jpn1	ALN43736.1	KT894734
Proteus mirabilis HI4320	CAR44921.1	AM942759
ICE*Spu*PO1	ABM23927.1	CP000503.1
ICE*Val*A056-1	ALJ83427.1	KR231688
ICE*Val*A056-2	ALJ83448.1	KR231689
ICE*Val*E0601	ALF34889.1	KT072768
ICE*Val*HN396	ALF35060.1	KT072770
ICE*Val*HN437	ALF35173.1	KT072771
ICE*Val*HN492	ALF34892.1	KT072769
ICE*Vch*ban5	ACV96187.1	GQ463140
ICE*Vch*Ban8	AFD29102.1	JQ345361
ICDC-143	ALP44393.1	KT151654
ICDC-956	ALP44487.1	KT151655
ICDC-1605	ALP44574.1	KT151656
ICDC-1627	ALP44665.1	KT151657

ICDC-1909	ALP44758.1	KT151658
ICDC-1944	ALP44847.1	KT151659
ICDC-2255	ALP44942.1	KT151660
ICDC-4210	ALP45128.1	KT151662
ICE of wujiang-2	ALP45323.1	KT151664
AHV1003	ALP45230.1	KT151663
VC1786	AEU11371.1	JN648379
ICE*Vch*ind5	ACV96374.1	GQ463142
ICE*Vch*mex1	ACV96456.1	GQ463143
ICE*Vfl*Ind1	ACV96519.1	GQ463144
ICE*Sh95*	WP_055647227.1	NZ_LGYY00000000.1
ICE*Sh392*	WP_028870222.1	QFDC00000000

Table S2.
Set amino acid sequences used for the phylogenetic analysis.

**Elements**	**SetC Protein ID**	**SetD protein ID**	**SetR Protein ID**	**Accession number**
SXT	AAL59693.1	AAL67891.1	AAL59692.1	AY055428
R391	AAM08050.1	AAM08085.1	AAM08038.1	AY090559
ICE of *Alteromonas mediterranea* MED64	AGP81135.1	AGP81136.1	AGP81141.1	NC_023045
ICE of *Alteromonas mediterranea* UM7	AGP96766.1	AGP96767.1	AGP96772.1	NC_021713
ICE*Pal*ban1	ACV96042.1	ACV96127.1	ACV96083.1	GQ463139.1
ICE*Pda*Spa1	CAQ35009.1	CAQ35010.1	CAQ35014.1	AJ870986.2
ICE*Pmi*Chn1	ALP69298.1	ALP69299.1	ALP69305.1	KT962845.1
ICE*Pmi*Jpn1	ALN43803.1	ALN43804.1	ALN43809.1	KT894734
Proteus mirabilis HI4320	CAR44802.1	CAR44800.1	CAR44790.1	AM942759
ICE*Spu*PO1	ABM24020.1	ABM24021.1	ABM24026.1	CP000503.1
ICE*Val*A056-1	ALJ83380.1	ALJ83365.1	ALJ83406.1	KR231688
ICE*Val*A056-2	ALJ83532.1	ALJ83533.1	ALJ83538.1	KR231689
ICE*Val*E0601	ALF34856.1	ALF34857.1	ALF34862.1	KT072768
ICE*Val*HN396	ALF35019.1	ALF35052.1	ALF35006.1	KT072770
ICE*Val*HN437	ALF35118.1	ALF35136.1	ALF35176.1	KT072771
ICE*Val*HN492	ALF34970.1	ALF34971.1	ALF34976.1	KT072769
ICE*Vch*ban5	ACV96169.1	ACV96200.1	ACV96204.1	GQ463140
ICE*Vch*Ban8	AFD28998.1	AFD28997.1	AFD28992.1	JQ345361
ICDC-143	ALP44315.1	ALP44314.1	ALP44309.1	KT151654
ICDC-956	ALP44402.1	ALP44401.1	ALP44396.1	KT151655
ICDC-1605	ALP44496.1	ALP44495.1	ALP44490.1	KT151656
ICDC-1627	ALP44582.1	ALP44581.1	n.i.	KT151657
ICDC-1909	ALP44674.1	ALP44673.1	ALP44668.1	KT151658
ICDC-1944	ALP44767.1	ALP44766.1	ALP44761.1	KT151659
ICDC-2255	ALP44856.1	ALP44855.1	ALP44850.1	KT151660
ICDC-4210	ALP45038.1	ALP45037.1	ALP45032.1	KT151662

ICE of wujiang-2	ALP45239.1	ALP45238.1	ALP45233.1	KT151664
AHV1003	ALP45137.1	ALP45136.1	ALP45131.1	KT151663
VC1786	AEU11456.1	AEU11457.1	AEU11462.1	JN648379
ICE*Vch*ind5	ACV96379.1	ACV96364.1	ACV96412.1	GQ463142
ICE*Vch*mex1	ACV96468.1	ACV96487.1	ACV96476.1	GQ463143
ICE*Vfl*Ind1	ACV96503.1	ACV96584.1	ACV96596.1	GQ463144
ICE*Sh95*	WP_055648504.1	WP_000210563.1	WP_000854920.1	NZ_LGYY00000000.1
ICE*Sh392*	WP_000566752.1	WP_000210563.1	WP_000854920.1	QFDC00000000

n.i.: not identified in the Genbank sequence

Figure S1

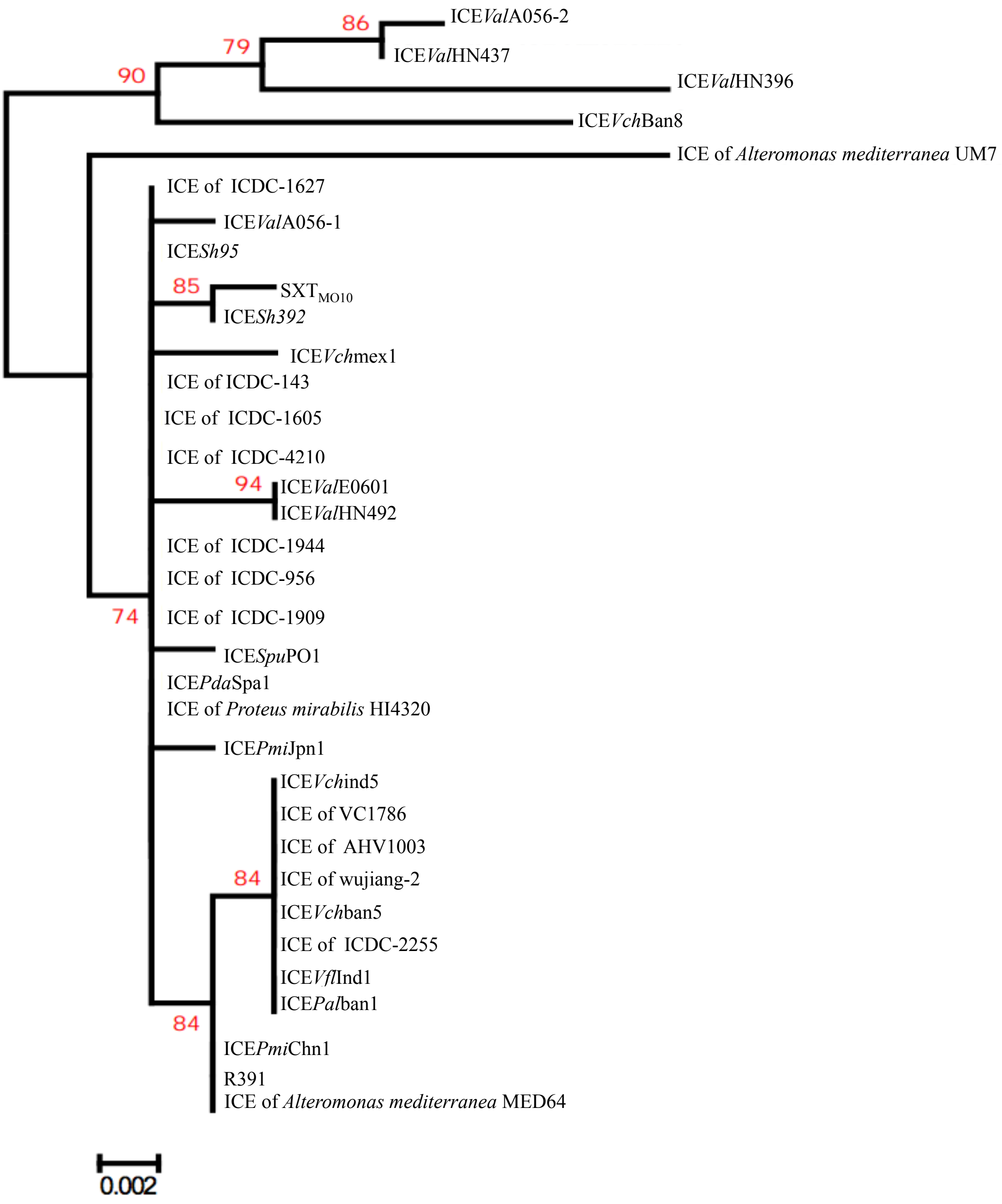

Figure S1. Phylogenetic tree of Set proteins was done using the maximum likelihood algorithm. SetC, SetD and SetR protein sequences were independently alingned using Clustal Omega v1.1.1 and concatenated. Tree construction was done using PHYML v3.1.

Figure S2

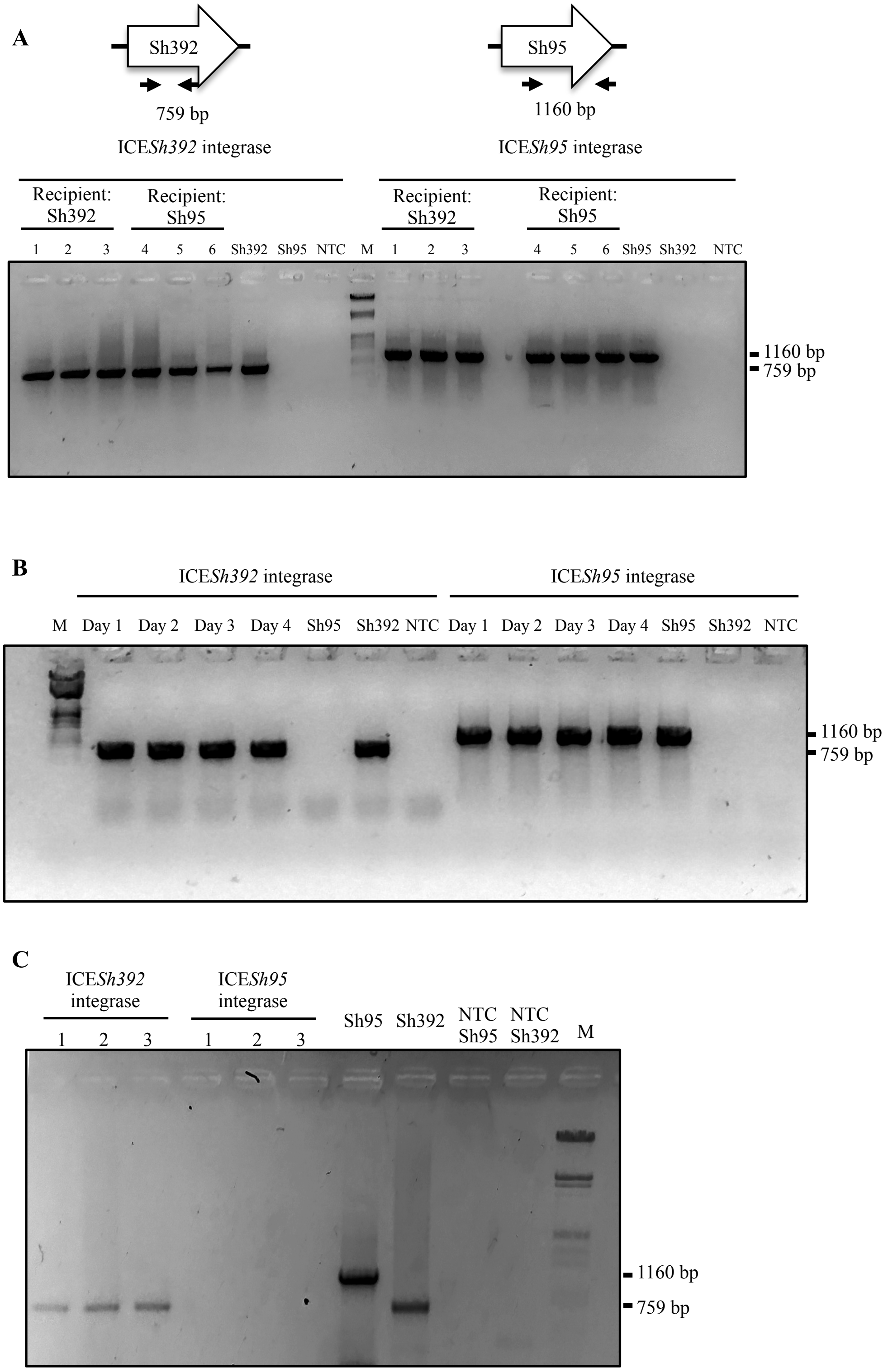

Figure. S2. A. Co-existence assay of ICE*Sh95* and ICE*Sh392* using strain *Shewanella* sp. Sh95 as donor and strain *S. algae* Sh392 as recipient and viceversa. Left scheme: detection of ICE*Sh392* integrase in all the samples. Right scheme: detection of ICE*Sh95* integrase in all the samples. Samples 1, 2 and 3 were obtained from the assay using strain Sh392 as the recipient cell whereas samples 4, 5 and 6 were obtained from the assay using strain Sh95 as the recipient cell. Sh95 and Sh392 correspond to positive PCR controls using the respective genomic DNAs. NTC correspond to the non template control of the PCR. M corresponds to the molecular marker. **B. Maintenance assay of ICE*Sh95* and ICE*Sh392* using strain *S. algae* Sh392 as host.** Agarose gel (1%) showing a representative result of the PCR assays done for detecting the integrases of ICE*Sh392* (left) or ICE*Sh95* (right) from genomic DNA extracted after serial growth during 4 consecutive days. **C. Re-transfer assay of ICE*Sh95* and ICE*Sh392* using *Shewanella* sp. Sh95::ICE*Sh392* as donor.** Samples 1, 2 and 3 correspond to three independent transconjugant colonies. Left: detection of ICE*Sh392* integrase. Right: detection of ICE*Sh95* integrase.

Figure S3

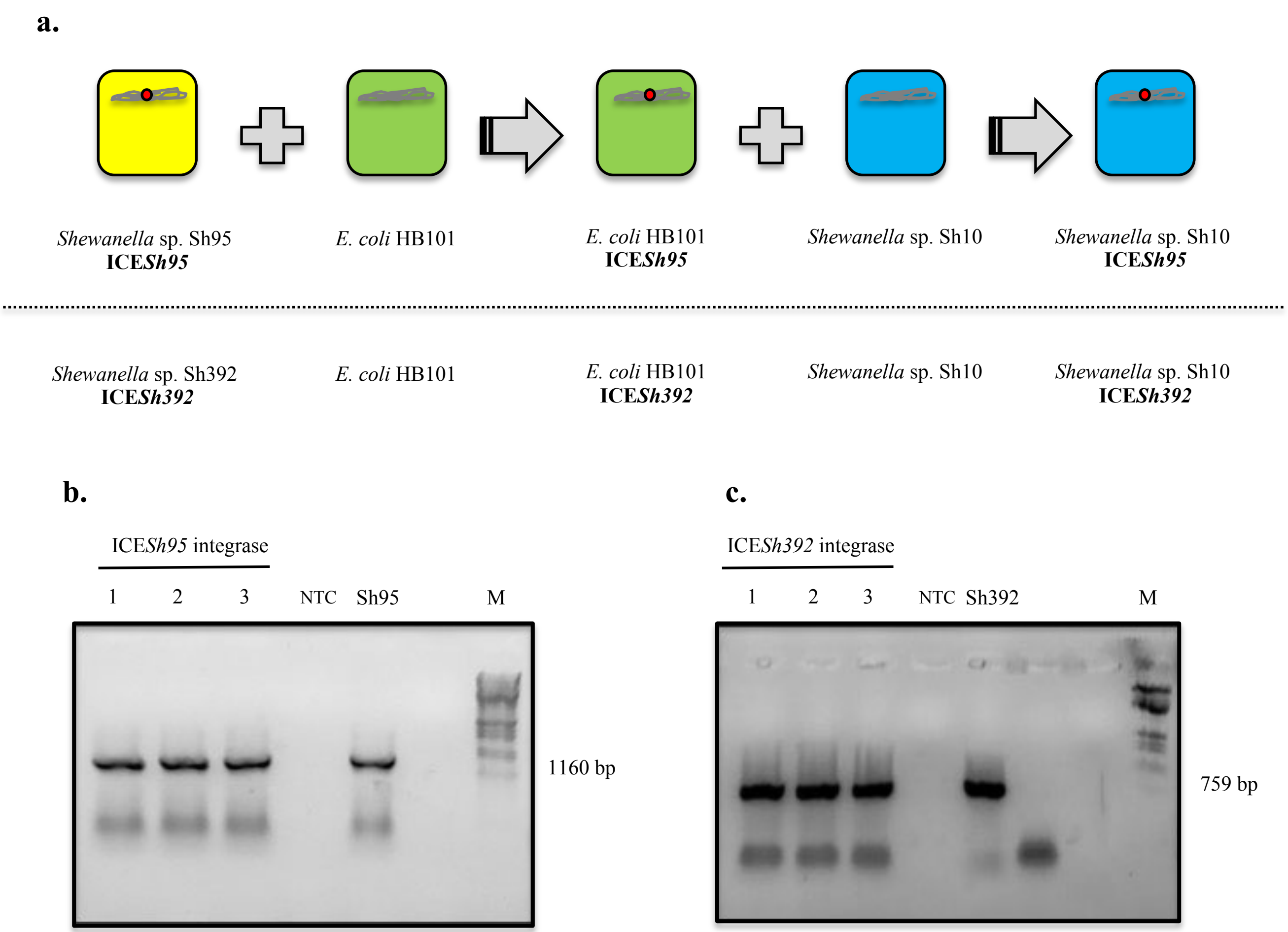

Figure S3. Re-transfer assays. A) Scheme depicting the subsequent mating assays. ICE*Sh95* or ICE*Sh392* (red circles) were transferred to *E. coli* HB101 (in green) from their respective hosts (in yellow). Once obtained each transconjugant (*E. coli* HB101::ICE*Sh95* and *E. coli* HB101::ICE*Sh392)* each ICE was conjugated to *Shewanella* sp. Sh10 (in light blue). **B)** Detection of ICE*Sh95* integrase gene by PCR in three independent colonies. **C)** Detection of ICE*Sh392* integrase gene by PCR in three independent colonies.
